# The Epidemiology of Animal-Associated Methicillin-Resistant *Staphylococcus aureus*

**DOI:** 10.3390/antibiotics12061079

**Published:** 2023-06-20

**Authors:** Martyna Kasela, Mateusz Ossowski, Ewelina Dzikoń, Katarzyna Ignatiuk, Łukasz Wlazło, Anna Malm

**Affiliations:** 1 Department of Pharmaceutical Microbiology, Medical University of Lublin, Chodzki Street 1, 20-093 Lublin, Poland; martyna.kasela@umlub.pl (M.K.); anna.malm@umlub.pl (A.M.); 2 Department of Animal Hygiene and Environmental Hazards, University of Life Sciences in Lublin, Akademicka 13, 20-950 Lublin, Poland; lukasz.wlazlo@up.lublin.pl; 3 Student’s Scientific Circle, Department of Pharmaceutical Microbiology, Medical University of Lublin, Chodzki Street 1, 20-093 Lublin, Poland; 55570@student.umlub.pl (E.D.); 57645@student.umlub.pl (K.I.)

**Keywords:** MRSA, *Staphylococcus aureus*, epidemiology, transmission, animals, antibiotic resistance, host adaptation

## Abstract

Methicillin-resistant *Staphylococcus aureus* (MRSA) remains an important etiological factor of human and animal infectious diseases, causing significant economic losses not only in human healthcare but also in the large-scale farming sector. The constantly changing epidemiology of MRSA observed globally affects animal welfare and raises concerns for public health. High MRSA colonization rates in livestock raise questions about the meaning of reservoirs and possible transmission pathways, while the prevalence of MRSA colonization and infection rates among companion animals vary and might affect human health in multiple ways. We present the main findings concerning the circulation of animal-associated MRSA (AA-MRSA) in the environment and factors influencing the direction, mechanisms, and routes of its transmission. Studies have shown it that *S. aureus* is a multi-host bacterial pathogen; however, its adaptation mechanisms enabling it to colonize and infect both animal and human hosts are still rarely discussed. Finally, we elaborate on the most successful strategies and programs applied limiting the circulation of AA-MRSA among animals and humans. Although MRSA strains colonizing animals rarely infect humans, they undergo host-adaptive evolution enabling them to spread and persist in human populations.

## 1. Introduction

*Staphylococcus aureus*, including methicillin-resistant *S. aureus* (MRSA), is not only one of the most common etiological factors of human infectious disease but also a pathogen with a profound impact on animal welfare because of increasing antibiotic resistance and the resulting economic burden [1,2]. The first reports concerning MRSA date back to the years 1959–1960, when it evolved from a susceptible *S. aureus* strain as a result of the implementation of methicillin as a new antimicrobial drug in hospitals. Additionally, the first observations suggested that it originated from one epidemic clone as all investigated strains exhibited the same antimicrobial resistance profile (penicillin, streptomycin, and tetracycline), [3]. Described by the Centers for Disease Control and Prevention as an note-threating microorganism quickly acquiring antibiotic resistance, MRSA gradually spread around the world, resulting in a high epidemiological burden both for humans as well as animals [4]. The strains are all characterized by the presence of an acquired type of resistance to β-lactam antibiotics encoded by genes from the *mec* group (*mecA*, *mecB*, and *mecC*) located in the staphylococcal chromosomal cassette (*SCCmec*). Genes encode for novel types of the penicillin-binding protein exhibiting decreased affinity to β-lactams resulting in inactivation of an antibiotic [5]. Depending on the setting, where the MRSA infection has been first reported, strains are divided into certain subpopulations. For human infections, the most prevalent hospital-associated MRSA (HA-MRSA) and community-associated MRSA (CA-MRSA) inform as to whether the strains were isolated in a nosocomial environment or outside, respectively. The third subpopulation—animal-associated MRSA (AA-MRSA), sometimes limited only to livestock-associated-MRSA (LA-MRSA)—includes pathogens isolated from animals [6,7]. The determination of subpopulation type can be helpful in epidemiological investigation by providing information about certain MRSA clone origins; however, the dynamic spread and transmission of MRSA worldwide blur the formerly clear line between clones of human and animal origin.

Since first MRSA was discovered, it has consequently gained resistance to other classes of antibiotics, like macrolides and tetracyclines, or chemotherapeutics, like fluoroquinolones, resulting in difficulties in the development of successful antimicrobial therapy in infected individuals [8]. While even more health-threatening *S. aureus* strains have emerged, including vancomycin-resistant *S. aureus* (VRSA) often characterized by multi-drug resistance, MRSA still remains the main therapeutic challenge worldwide [9]. Depending on the character and location of infection, routine antimicrobial therapy of MRSA infections consists of the application of several antimicrobial drugs, i.e., vancomycin in the case of MRSA bacteremia, daptomycin in the treatment of soft-tissue infections, or mupirocin for skin infections. For infections caused by multi-drug-resistant strains, novel antimicrobial agents are often applied, e.g., linezolid antibiotic, or multiple semisynthetic drugs, such as tigecycline, dalbavancin, oritavancin, iclaprim, cethromycin, or delafloxacin [10]. Except standard antibiotic therapy, alternative treatments and agents supporting antibiotic therapy are being developed as a new approach for the treatment of MRSA infections. The above-mentioned strategies consist of combining known antimicrobial agents with substances or compounds of natural origin or the application of a combination of two or more antimicrobial agents [8]. In spite of all these efforts, the emergence of resistance to new antimicrobials is being observed. MRSA strains resistant to daptomycin were described just years after the official Food and Drug Administration approval for the treatment of *S. aureus* in 2006. A similar situation has been noted for linezolid, implemented for treatment in the year 2000, to which resistance was first described in 2005. Since then, *S. aureus* has developed several resistance mechanisms [11].

The growing antibiotic resistance to so called “last resort” antibiotics in the treatment of serious bacterial infections drastically limits the current therapeutic options. There are several factors that increase the risk of acquiring antibiotic resistance in bacteria. They include: the excessive use of antimicrobial agents creating selective pressure enabling resistance to develop and persist in the environment, the emergence of novel sources of drug-resistant bacteria as well as novel routes for these bacteria to spread which allows not only the direct transmission of MRSA but also the occurrence of the horizontal transfer of resistance factors [12]. In the recent years, animals have become a profound secondary source of MRSA in the environment, and the frequent contact between animals and humans create a significant route of their transmission [13]. Therefore, to further fight antibiotic resistance in *S. aureus*, it is indispensable to investigate and understand novel reservoirs of antibiotic-resistant bacteria and the evolutionary consequences of their global spread. The epidemiology of MRSA is changing dynamically due to intensive circulation within the community and farming environment [14]. However, the transfer of microorganisms between humans and animals seems to be a part of the natural process of microbial adaptation. The interspecies transmission of antibiotic-resistant bacteria raises significant concerns for public health. The prevalence and risk factors for colonization and subsequent infection with multidrug-resistant microbes among humans are well established when compared with animal populations [15]. MRSA colonizes and infects companion animals and wild animals as well as livestock, causing serious diseases of worldwide significance, e.g., in poultry or dairy cows [16].

In the present day, routine epidemiological investigation of MRSA includes the application of a variety of genotyping techniques, enabling interfacility results comparison. The most prevalently used multi-locus sequence typing (MLST) provides information about the clonal complexes (CC) and sequence types (ST) of MRSA circulating in the environment. A steady decrease in the costs of whole genome sequencing (WGS) and the development of techniques enabling high-throughput analysis have resulted in novel research in the field of microbial transmission [17]. Recent findings concerning the spread of MRSA in the environment provide valuable data useful in the development of control strategies and successful measures significantly limiting AA-MRSA transmission.

In spite of multiple advancements in the treatment of bacterial infections, multi-drug-resistant strains of *S. aureus* undeniably remain one of the major pathogens in animals and humans. This review not only summarizes crucial information concerning the most important characteristics of AA-MRSA but also points out the main findings about its spread in the environment and the possibility and mechanisms of its transmission between various hosts, taking into account the interconnection between animals and humans. Finally, this review is aimed at discussing the most successful strategies applied to limit the circulation of MRSA in the animal farming environment, creating simple guidelines and presenting general recommendations.

## 2. Methicillin-Resistant *Staphylococcus aureus* in Companion Animals

Companion animals might be a significant reservoir of MRSA circulating in the environment. The presence of multi-drug-resistant bacterial strains in households poses a threat not just to human and animal health but especially for people who are immunocompromised because of various medical conditions as well as those undergoing immunosuppressive treatment. Therefore, epidemiological investigations on MRSA colonization rates in companion animals provide valuable data on the scale of the problem.

Studies have shown that the nares, mouth, and perineum are the major colonization sites in cats and dogs; however, the persistence of carriage itself remains poorly investigated [18]. Both animals and owners can be colonized by *S. aureus* as an effect of indirect everyday interaction with each other as well as contact with contaminated surfaces within the household [19,20]. Multiple studies have detected the presence of MRSA in not only pets, mainly dogs and cats [21,22,23,24,25,26,27,28,29], but also other companion species like birds, guinea pigs, turtles [30], or hamsters [31]. This wide dissemination proves that MRSA is well adapted to colonize a wide spectrum of animal hosts.

The methicillin-resistant *S. aureus* colonization rates given by multiple studies are highly diversified and depend on various factors, including geographical location, the animal population studied, household hygienic conditions, and many others. Recent studies have shown alarmingly high MRSA colonization rates in most common species of companion animals—dogs and cats. Moreover, taking into consideration the number of cats and dogs kept as companion animals worldwide, the scale at which transmission between pets and the owners might occur is disturbing. In the study conducted by Strommenger et al. in Germany, all *S. aureus* strains isolated from pet dogs and cats harbored the *mecA* gene [32]. Similarly, relatively high MRSA colonization rates were observed in France, where MRSA colonized 39.3% of dogs, 26.5% of cats, and as high as 47.1% of horses [25]. Also, Drougka et al., whose study was located in Greece, investigated the prevalence of *S. aureus* among companion dogs and cats and found 37% and 30% methicillin-susceptible *S. aureus* (MSSA) isolation rates, respectively, while the overall MRSA prevalence rate accounted for 10.8% [33]. The prevalence of certain clonal lineages of MRSA isolated from companion animals remains similar within European countries and, according to the authors, often reflects dominating lineages of MRSA of human origin (Table 1). In contrast to dogs and cats, only horses are usually colonized by MRSA strains typical for livestock, e.g., in France, as many as 72.1% of MRSA strains isolated from horses belonged to CC398 [25].

Fewer researchers have focused on the possibility of interspecies transmission between pets and their owners and conducted studies with the use of genotyping methods [22]. Researchers suggest that transmission from humans to companion animals occurs more prevalently due to overlapping of their habitats [46]. Moreover, multiple studies have proven that humans are the main source of the MRSA colonizing companion animals, which would explain the high colonization rates in cats and dogs maintaining close contact with owners and living in an area of limited space. These animals might become a profound secondary source for human and animal infections, which is emphasized by the fact that strains of human origin, especially HA-MRSA, often carry more antibiotic-resistance and virulence genes than strains of animal origin [25]. The reports of typical nosocomial MRSA strains’ isolation from dogs and cats (e.g., ST5, ST45, and ST239) prove that pets might act as a secondary reservoir for virulent *S. aureus* strains in the environment [22]. The enhanced virulence of MRSA is often connected with the production of specific toxins resulting in more severe disease symptoms in the case of infection. The production of Panton–Valentine leucocidin toxin, strongly associated with skin and soft-tissue infections and tissue necrosis in community-acquired pneumonia, might have serious health implications not only for immunocompromised people but also for young and healthy individuals [47]. The high isolation rates of *pvl*-positive *S. aureus* strains from healthy dogs and cats in certain European regions, ranging from 25% to even 87.5%, underlining the need for epidemiological monitoring of MRSA colonizing pet animals, especially in the context of pet owners predisposed to community-acquired staphylococcal infections [24,33,48].

The main concern about *S. aureus* transmission between humans and animals is the spread of zoonotic diseases in the general population; nevertheless, a recent study conducted by Bierowiec et al. proved that close contact with owners predisposed companion cats to significantly higher *S. aureus* colonization rates than free-living, domestic cats. Moreover, the prevalence of MRSA was also found to be higher among pet cats, which confirms the assumed direction of MRSA transmission from owners to companion animals [21]. Other factors recently discovered to be significantly associated with the *S. aureus* colonization of both dogs and cats are the young age of the animals (<12 mo.), living in rural areas, possessing skin diseases at the time of swab collection, and simultaneous colonization with coagulase-negative staphylococci [33].

Recent studies clearly show the growing importance of companion animals as a secondary reservoir of drug-resistant pathogens in the environment. Human infections caused by MRSA isolated simultaneously from companion animals occur rarely; however, researchers emphasize the role of proper hygienic conditions in households in limiting the risk of colonization and subsequent infection in pet owners [23,31].

## 3. Methicillin-Resistant *Staphylococcus aureus* in Livestock Animals

The human population is steadily expanding into new geographical areas, which together with the increase in the number of large-scale animal farms generates new transmission pathways that ease the spread of MRSA in the environment. High MRSA colonization rates in livestock farming environments and the emergence of LA-MRSA in humans raise questions regarding its origin and possible transmission pathways [49]. Similar to companion animals, MRSA colonizing livestock can act as a significant reservoir for drug-resistance genes. The transmission of these genes is a significant epidemiological concern, because of the possible share in acquiring MRSA that colonizes humans. The high diversification of LA-MRSA isolation rates and their genetic variants in animal farming environments is common and clearly seen in studies from different parts of the world.

Researchers suggest that the current spread of LA-MRSA in Europe is connected to the international pig market [50]. What is more, the prevalence of LA-MRSA in pigs is rising constantly, with ST398 LA-MRSA lineage domination observed in most European countries [51,52]. Despite this, the epidemiological situation in other parts of the world differs. The domination of certain lineages of LA-MRSA in livestock farming environments is being observed (Table 2). The distribution of MRSA sequence types among livestock depends not only on geographical location [53,54] but also the major clonal lineages causing infections in humans, e.g., in Australian piggeries, as many as 84% of MRSA strains were classified as ST93—the most common CA-MRSA in the country. Additionally, MRSA isolation rates were high and accounted for 76% in animals and 60% in pig farm workers [55].

*Staphylococcus aureus* remains a major etiological factor of bovine mastitis [67,68], and it is estimated that methicillin-resistant strains are responsible for approximately 12% of infections [69,70]. Recent findings confirm that MRSA strains circulating among humans are capable of causing infection in cows [71]. Juhász-Kaszanyitzky et al. found that subclinical mastitis in cows on a Hungarian farm was caused by MRSA genetically undistinguishable from a strain isolated from a farm worker. Moreover, an alarmingly high percentage of MRSA strains isolated from dairy cows harbor multiple enterotoxin genes simultaneously (*seg*, *sei*, *sem*, *sen*, *seo*, and *seu*), making a possible outbreak from contaminated milk more health-threatening to humans [61].

Methicillin-resistant strains are also present in poultry [49,57,58,72] and poultry-derived products [73,74]. In some countries, the prevalence of MRSA among poultry has been found to be relatively high. In Algeria, as many as 57% of laying hens and 50% of broiler chickens were found to be colonized with MRSA. The authors also found that the poultry were significantly more often colonized than the bovine animals (31%), [49]. The poultry were found to be colonized not only by livestock-associated CC398 but also by strains of human origin [58]; thus, the epidemiological situation regarding poultry market should be carefully monitored in order to limit the spread of virulent MRSA strains in the environment.

In comparison to the environment of large-scale farms with bovine, swine or poultry, the epidemiology of *S. aureus* differs in small dairy-ruminant herds. Carfora et al. observed low intra-farm prevalence of both MSSA and MRSA among sheep. In total, 2.16% of milk samples were found *S. aureus* positive and only 0.34% MRSA positive, however, genotyping revealed that all MRSA collected from animals and farm workers belonged to the same MLST variant (ST1), [75]. MRSA is also being isolated from goats. Loncaric et al. described a case of necropsy in a goat caused by LA-MRSA ST398. The same strain was isolated earlier from the goat’s owner, proving the infectious potential of LA-MRSA transmitted from the human host to the animal [76]. In the Czech Republic, LA-MRSA ST398 was isolated on a goat farm, both from animals and personnel, indicating circulation of *S. aureus* in a given environment; however, the authors did not detect any MRSA strains among sheep and pig farms in the same homestead [62]. The presence of MRSA was also found in rabbits; *S. aureus* was detected both as a colonizing agent as well as an etiological factor causing lesions. Large-scale studies in commercial rabbitries located on the Iberian Peninsula revealed the presence of MRSA in 19 out of 89 farms with an 11.25% colonization rate among rabbits [63].

The presence of MRSA in the large-scale farming environment challenges current epidemiological approaches limiting the circulation of pathogens between animals and humans. Moreover, it increases the probability of the emergence of new pathogenic microorganisms, making agro-ecosystems a global threat to public health.

## 4. Transmission of Animal-Associated *Staphylococcus aureus* between Animals and Humans

Transmission of pathogens between animals and humans has been occurring since humans develop farming practices that made them stay in close contact with animals. Current epidemiological studies report a growing rate of infections in humans caused by pathogens of animal origin. In the last decade, the main methicillin-resistant livestock-associated clonal lineage, ST398, has been isolated more recently from humans. Its presence has been detected in colonized individuals having direct contact not only with farming environments but also in nosocomial environments, where it is an etiological factor of a wide range of infections. The alarming emergence of ST398 in hospitals can further ease its spread and thus help it to persist in the human population as an effect of human-host adaptation [77]. Recently, the presence of human-adapted ST398 MRSA strains in clinical samples in Taiwan has been reported [78]. What is important is that ST398’s emergence as an etiological factor of human infections in Europe results from the intensive export of pigs from countries with a high ST398 burden in the farming environment, e.g., the Netherlands [50].

Large-scale survey studies that made a collection of data available reflecting the current epidemiological situation concerning the dynamic of the spread of livestock-associated MRSA strains among humans revealed increasing trends in LA-MRSA isolation rates from human clinical samples. Livestock-associated strains of MRSA were detected among humans in 17 out of 19 EU countries, with the highest LA-MRSA prevalence rates noted for the Netherlands (30.7%), Denmark (29.3%), and Spain (9.7%), [79]. The prevalently found bovine strains CC97 is considered as an emerging etiological factor of human infection. This observation is confirmed by a statistically significant increase in the share of CC97 among *S. aureus* isolated from human infections in Denmark over a five-year period (from 0.3% in the year 2007 to 1.7% in 2011), [80]. Although we did not find any research covering the same topic that is more recent, this study demonstrates an important trend concerning CC97 epidemiology. Other authors estimated the LA-MRSA burden among the human population in Eastern England classifying the prevalence of LA-MRSA among MRSA-infected patients as low (less than 1%), [16]. Although most of the studies mentioned investigated the occurrence of LA-MRSA among humans in European countries, these strains have also been isolated in the US [66,81], Canada [82], China [78,83], and India [84].

Whereas advanced genotyping methods allow for the genetic comparison of *S. aureus* of human and animal origin, it is still difficult to undeniably prove the source of MRSA and the direction of its transmission [71]; thus, studies should cover longer periods of time along with repeated sampling [46,85]. The Denmark region has one of the most thoroughly investigated epidemiological situations concerning LA-MRSA strains [86,87]. Studies revealed that in Denmark, the increased prevalence of MRSA colonization and infection rates among pigs as well as humans was caused by the clonal spread of only a few lineages of CC398. Moreover, moving animals from farm to farm remained the main route of MRSA transmission [88]. An alarming situation might be found in countries where pigs are considered to be the main reservoir for MRSA, which in addition to a low prevalence of MRSA of human origin in the nosocomial environment creates a substantial epidemiological problem concerning the transfer of MRSA from livestock to the general community. Additionally, many authors have proven that people working in a farming environment are at higher risk of livestock-associated CC398 colonization [56].

The role of wild animals in intra- and interfarm MRSA transmission is a matter rarely discussed [89]. Methicillin-resistant *S. aureus* strains have already been detected in multiple species of wild animals, e.g., boars, deers, hares, or hedgehogs [90,91,92,93,94]. Despite the wide genetic diversity of isolated MRSA, typical LA-MRSA strains like ST398 have also been detected. A recent study indicated the possibility of MRSA transmission on a Canadian pig farm via colonized Norway rats. In spite of the fact that the prevalence rates of MRSA colonization in rats are currently classified as low, this route of MRSA dissemination should be considered in large-scale epidemiological studies [95,96].

The transmission of MRSA between species might also occur indirectly—via contaminated air and dust particles [97]. Locatelli et al. noticed the presence of the same genetic variants of LA-MRSA among dairy cattle, swine, farm workers, and dust samples, suggesting the wide strain dissemination in the environment and indicating the complexity of its transmission as well as great potential for zoonotic transmission [60]. A Danish study concerning airborne MRSA on pig farms revealed that MRSA was present in particles of different sizes enabling inhalation and deposition not only in the human upper airways but also in the bronchi and the alveoli. The authors found that 21.5% of *S. aureus* present in the air was able to deposit in the primary and secondary bronchi [98]. The airborne colonization models proved that the concentration of airborne MRSA significantly influences the percentage of MRSA-positive environmental swab samples. What is more, the concentration of MRSA in the air affects the duration of colonization among piglets. A recent study showed that, dependent on the MRSA concentration, animals were colonized permanently (10^6^ CFU/m^3^), transiently (10^4^ CFU/m^3^), or non-colonized (10^2^ CFU/m^3^), [99].

The complexity of human–animal interactions as well as the abundance of transmission routes not only intensify MRSA circulation but also make an epidemiological investigation a lot more demanding and require the use of advanced genotyping methods.

## 5. Host-Adaptation Mechanisms

One of the greatest concerns of modern epidemiology of infectious diseases is the ability of animal pathogens to adapt to the human host. Many studies had proven that *S. aureus* is a multi-host bacterial pathogen; however, the mechanisms thanks to which this bacterium achieved this ecological success are rarely studied. *S. aureus* strains usually harbor a specific pool of genes, allowing for host-adaptation; however, only a few combinations provide a universal package, providing the capacity to colonize and infect multiple host species [100]. Although various MRSA clonal complexes are being isolated from large-scale animal farming environments, only a few of them seem to have an ability to spread and persist among animal populations. A large-scale study concerning MRSA epidemiology on Norwegian farms proved that MRSA variants within CC1 had the ability to adapt and persist in a swine population for longer periods of time, which is connected with the changing epidemiology of LA-MRSA [101]. Researchers also investigated the capability of MRSA strains of human origin commonly colonizing animals to readapt back to human host. Everyday contact between companion animals and their owners might trigger readaptation leading to the spread of MRSA strains among humans [25].

**Figure 1:**
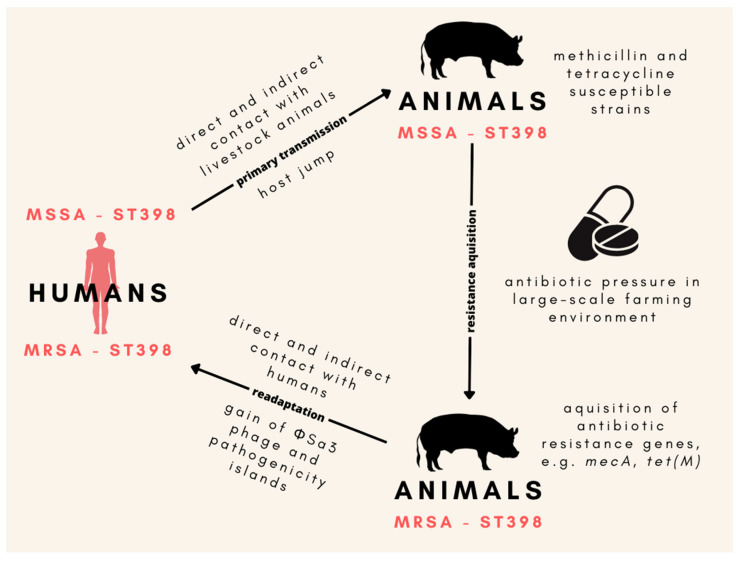
The origin of the ST398 MRSA lineage in animals and the main mechanisms of its readaptation to human hosts. Animal-associated MSSA ST398 developed as a result of its transmission from humans to animals, which was followed by the gain of methicillin and tetracycline resistance induced by the presence of antibiotics in the large-scale farming environment. Then, MRSA ST398 stabilized in the animal population. Close contact between humans and animal farms led to the readaptation of MRSA ST398 back to the human host, which was enabled by the acquisition of the ΦSa3 prophage carrying the human innate immunomodulatory genes. MSSA—methicillin-susceptible *Staphylococcus aureus*; MRSA—methicillin-resistant *S. aureus*.

One of the main components of the *S. aureus* genome allowing for adaptation to the human host is the presence of the ΦSa3 phage encoding proteins responsible for the ability to avoid the response of the innate immune system. Researchers have observed that the human to livestock host jump of *S. aureus* is often connected to the loss of the above-mentioned genetic element [102]. Moreover, ΦSa3-positive variants of LA-MRSA strains have been reported in chicken meat products; thus, poultry meat might be a significant source of LA-MRSA strains in the community, posing a health threat to people without direct contact with the farming environment [103]. Novel data provide strong proof that LA-MRSA ST398 originally evolved from human strains and then established in animal populations along with the loss of ΦSa3 prophage carrying the human innate immunomodulatory genes (Figure 1). Primary methicillin and tetracycline-susceptible ST398 acquired resistance after host switch, which additionally, raises serious questions regarding the role of the selection of antibiotic-resistant strains as a result of the extensive use of antibiotics in animal production [104]. Currently, one of the main features distinguishing LA-MRSA CC398, from CA- and HA-MRSA strains of human origin is that animal-associated clones often harbor the *tet(M)* gene and are therefore resistant to tetracyclines [25,105].

The presence of staphylococcal pathogenicity islands might also be involved in a host range of various clonal lineages of MRSA. This mobile structure contains important virulence factors enabling colonization and infection within different types of tissue [106]. For example, novel pathogenicity island SaPI-S0385 encodes extracellular factors like the von Willebrand factor binding protein and staphylococcal complement inhibitor that favor the development of infection in humans [107]. Although large genetic variations among MRSA lineages are common, strains exhibiting host-specific features have been observed [108]. Bacteriophage phi3 gene cluster encoding immune evasion proteins have been isolated more frequently in MRSA isolated from humans than from animals [109]; however, a recent longitudinal study downgraded its role as an evolutionary driver in the readaptation of AA-MRSA to human hosts [106]. Some studies revealed that certain genetic variants of the HA-MRSA USA300 lineage are able to persist within keratinocytes, simultaneously evading clearance and leading to skin infections in humans [110]. As mentioned above, the presence of certain genes connected with adaptation to human hosts has been investigated and described; however, the situation remains largely unknown when it comes to the animal adaptation of MRSA. Additionally, future large-scale studies should focus on determining the universal package of genes encoding proteins that provide adaptation to multiple hosts resulting in the evolutionary success of major clonal lineages of MRSA.

Studies relying on comprehensive phylogenetic analysis of AA-MRSA reveal novel insights about its host-adaptation mechanisms. Richardson et al. revealed that humans are a major donor of *S. aureus* to pigs, cows, sheep, goats, carnivores, birds, rabbits, horses, and rodents. Authors have also observed some interesting host-switching trends. Cows were considered as the most prevalent recipient for human *S. aureus* and the strains of sheep origin were less likely to spread to other animals [100]. Sakwińska et al. showed the patterns of one-directional host adaptation of *S. aureus* between farmers and dairy cows. Over one-third of *S. aureus* strains isolated from mastitis belonged to the widely recognized human complex CC8. The authors explained this unusual observation by progressing bovine adaptation of typically human *S. aureus* occurring in Switzerland and European regions located nearby. On the other hand, the low rate of bovine-to-human transmission in the same environment suggested the loss of genetic determinants, allowing for the colonization of humans [67]. Taking into account close genetic relationships between humans and monkeys (non-human primates), researchers investigated the possibility of *S. aureus* transmission between these two groups of mammals [46,111]. Senghore et al. proved that the direction of transmission from human to non-human primates (green monkeys) was occurring more frequently. Moreover, WGS of isolates of human and monkey origin showed that the human-to-monkey host switch occurred nearly 3000 years ago and relied on the loss of genes allowing for human colonization [111].

Although livestock-to-human host shifts rarely occur, they might have an important meaning for public health. Some authors proved that a few of the LA-MRSA strains were not only able to switch hosts and spread among humans but were found as pandemic etiological factors of infection among people [80]. The line between human and animal-associated MRSA is consecutively blurring as an effect of the continuous and intense circulation of MRSA in the environment. For that reason, efforts should be made to limit the possible transmission pathways between humans and animals by implementing successful control and prevention programs.

## 6. Control Strategies and Monitoring Programs

The most effective action limiting the spread of zoonotic diseases is the implementation of strategies controlling their spread as well as creating successful programs enabling control of the current epidemiological situation. The prevalence of MRSA infection rates is rising both in livestock as well as in the general community along with the changes in the pathways and dynamics of MRSA transmission being observed on a global scale [112,113]. The spread of MRSA strains from animal farms to the general community might lead to an increase in colonization, carriage, and infection rates among humans. To limit the circulation of LA-MRSA between farms and the community, many programs have been established and strategies applied, some of them successful [112]. The influence of short-term contact with the animal farming environment on subsequent colonization and its duration among humans is being discussed. Angen et al. found that the nasal carriage of LA-MRSA was positively correlated with short-time exposure to MRSA present in the air as well as with active work on a swine farm. Despite the fact that 94% of people were MRSA-positive in their nasal cavity directly after exposure, colonization was classified as intermittent and lasted up to 48 h only [112]. The results suggest that reducing the time spent in direct contact with livestock animals and their environment can significantly limit the risk of AA-MRSA transmission to humans. Some authors suggest that a safe distance between farms might be a successful biosecurity strategy limiting the circulation of LA-MRSA between different animal species [60]. Another strategy that can be implemented in order to avoid the spread of AA-MRSA to humans is the active decolonization of farm workers, which is a part of the most unique and complex MRSA control strategy in swine farms applied in Norway. The program consists of routine annual screening of the swine population, followed by a more detailed examination with the implementation of control measures and eradication in the case of LA-MRSA detection, both among animals as well as farm workers [101]. Other strategies consist of actions preventing MRSA introduction into farms. Researchers suggest that the combination of multiple biosecurity protocols can efficiently limit the transmission of MRSA, e.g., disinfection of transport vehicles, purchase of MRSA-negative herds of animals, temporary quarantine of new herds, or limitations concerning farm workers, like showers before and after visiting farms or wearing gloves [114].

Except for the direct isolation of MRSA from animals, it is often detected in the environment, especially farm dust. Dust particles suspended in the air ease the deposition of LA-MRSA in the nasal cavity of animals and workers enabling its transmission. The role of this factor in LA-MRSA dissemination can be limited by applying efficient ventilation along with air filtration. On the other hand, high-pressure cleaning and intensive air movements lead to a rapid increase in airborne MRSA making the use of personal dust masks indispensable [98]. Studies have proven that airborne MRSA acquisition can be successfully reduced by wearing protective masks—only 9% of the participants wearing masks were LA-MRSA positive in their nasal cavity when compared to as much as 63% of unprotected short-term visitors [115]. These simple protection activities can significantly limit LA-MRSA circulation and transmission between humans and animals as well as reduce MRSA colonization rates. Additionally, the implementation of programs controlling the use of antibiotics can limit the emergence of new MRSA strains of animal origin in the large-scale farming environment [116]. Because studies have revealed that humans are the donors of the currently dominating MRSA strains in farming environments, efforts should be made to limit the contact of animals with humans, as well as the overlapping of living habitats of animals and humans. What is important is that such activities might have a positive effect both for animals and humans. Moreover, taking into account the intensive export and import of animals, especially pigs, international monitoring programs should be applied to gain detailed knowledge of the current epidemiological situation.

Except for standard procedures limiting the spread of MRSA in the environment, there are multiple interesting alternatives, including the application of probiotics, mainly lactic acid bacteria such as *Lactobacillus* spp., to reduce the MRSA colonization load in animals, or therapies using bacteriophages in the treatment of staphylococcal infections [114]. Although these strategies are promising, more comprehensive clinical studies are needed to evaluate their usefulness in vivo.

Although current research focuses mainly on the development of programs aiming at limiting the spread of MRSA in farming environments, the transmission of MRSA between owners and pets remains a significant matter, especially for immunocompromised people. Currently, the best practices recommended that might decrease the risk of MRSA transmission include maintaining basic hygienic activities in households with pets, washing hands before and after contact with an animal, or excluding access to certain spaces, e.g., the bedroom [117].

## 7. Conclusions

The transmission of AA-MRSA strains among livestock and farmers occurs via direct contact between animals and people as well as indirect contact with farm dust or contaminated equipment. Because multiple studies have detected the presence of the same genetic variants of AA-MRSA simultaneously in animals, farm workers, environmental samples, meat products, and milk (LA-MRSA has been isolated from a variety of sources), the determination of the direction of transmission is difficult; thus, researchers should focus more on the persistence of MRSA carriage and colonization and infection patterns. Most of the studies proved that *S. aureus* strains colonizing animals rarely infect humans. Nevertheless, AA-MRSA strains undergo host-adaptive evolution allowing them to spread and persist among human populations. Therefore, strategies to reduce the risk of emerging new pathogens of zoonotic origin should rely on surveillance conducted worldwide followed by the implementation of protocols as well as biosecurity measures limiting the spread of AA-MRSA in the livestock and community environment.

## Figures and Tables

**Table 1:** Metihicillin-resistant *Staphylococcus aureus* lineages isolated from pet animals.

Country	Years of Isolation	Animals	MLST	Literature
Germany	2003–2004	Dogs and cats	CC22 (ST22)	[32]
	2010–2012		CC22, CC5, CC398, CC8	[34]
England	2007–2008	Dogs	CC30 (ST36)	[35]
		Cats	CC22	
		Horses	CC22, CC8 (ST239)	
Switzerland	2017	Horse	ST338	[36]
Hungary	2017	Horse	ST130	[37]
France	2010–2015	Dogs	CC8, CC398, CC5, CC22, CC45, CC1, CC59	[25]
		Cats	CC8, CC398, CC5, CC22, CC130	
		Horses	CC398, CC8, CC130, CC49	
Serbia	2016	Dogs	CC239, CC45, CC5	[22]
		Cat	CC1	
Portugal	1999–2018	Dogs	CC22 (ST22), CC1 (ST188, ST6565, ST1), CC8 (ST72, ST6566), CC5 (ST105, ST5, ST6535), CC398 (ST398)	[38]
		Cats	CC22 (ST22), CC8 (ST72), CC97 (ST97), CC7 (ST7), CC15 (ST15), CC5 (ST5), CC398 (ST398)	
		Rabbits	CC22 (ST22), CC121 (ST121), CC398 (ST398)	
		Horse	ST816	
US	2010	Dogs, cats, and a hamster	CC5	[31]
Brazil	2010	Cat	ST30	[39]
	2010–2013	Dogs	ST1, ST5, ST30, ST239	[40]
Japan	2016–2018	Cats	ST5, ST764, ST512, ST1863, ST1, ST2725, ST4775, ST4779, ST8, ST4224, ST4777	[41]
		Dogs	ST72, ST4776, ST5, ST4778, ST2725, ST8, ST380	
		Rabbits	ST4768	
Australia	2019	Dogs	ST1, ST5, ST72, ST93	[42]
Thailand	2017–2020	Dogs	ST398, ST5926, ST6563	[43]
		Cats	ST398	
Malaysia	2007–2008	Dogs	ST59	[44]
		Cats	ST55	
Zambia	2012	Dogs	ST 152, ST398, ST15, ST1416	[45]

**Table 2:** Methicillin-resistant *Staphylococcus aureus* lineages isolated from livestock animals.

Country	Years of Isolation	Animals	MLST	Literature
Denmark	2004–2007	Pigs	ST398	[56]
	2013–2014	Ducks and turkeys		[57]
Belgium	2011	Chickens	ST398, ST239	[58]
Switzerland	2017	Pigs	ST398	[36]
Spain	2009–2010	Pigs	ST398	[51]
	2017–2018			
Portugal	2020	Quails	ST398, ST6831	[59]
Italy	2010	Pigs	CC398, CC97	[60]
	2018	Dairy cows	CC22	[61]
Czech Republic	2017	Goats	ST398	[62]
Spain and Portugal	2014–2017	Rabbits	ST2855, ST146, ST398	[63]
Australia	2015	Pigs	ST398, ST93, ST30	[55]
China	2011–2016	Pigs	ST9, ST764	[54]
		Chickens	ST9	
		Ducks	ST9, ST398	
	2017–2018	Pigs	ST9, ST3653-6356, ST1376, ST59, ST398	[64]
India	2020	Goats	ST772, ST22, ST368	[65]
Africa (Cote d’Ivoire)	2012	Goats	ST15, ST152, ST6, ST8, ST1472	[46]
		Sheep	ST15, ST88, ST152, ST567, ST121	
		Chicken	ST152	
USA	2006	Pigs	ST398	[66]
	2013–2014		ST9, ST398, ST2007, ST1, ST5	[53]
